# Sociodemographic correlates of food habits among school adolescents (12–15 year) in north Gaza Strip

**DOI:** 10.1186/1471-2458-9-185

**Published:** 2009-06-15

**Authors:** Abdallah H Abudayya, Hein Stigum, Zumin Shi, Yehia Abed, Gerd Holmboe-Ottesen

**Affiliations:** 1 Section for Preventive Medicine and Epidemiology, Institute of General Practice and Community Medicine, University of Oslo, Oslo, Norway; 2 Norwegian Institute of Public health, University of Oslo, Norway; 3 School of Public Health in Gaza, Al Quds University, Gaza City, Palestine; 4 Institute of General Practice and Community Medicine, University of Oslo, Oslo, Norway

## Abstract

**Background:**

There is little information about meal patterns and food consumption of adolescents in Palestine. The objective of this study was to describe the association between sociodemographic factors and food intake, and meal patterns among Palestinian school adolescents (12–15 year) in North Gaza Strip.

**Methods:**

A cross-sectional study was conducted in 2002 comprising 944 subjects in 10 schools in Gaza city, Jabalia village and Jabalia refugee camp. Self-administered questionnaires were filled in by students and parents to obtain data on frequency of meals, food intake and sociodemographic characteristics.

**Results:**

High household socioeconomic status (SES) was associated with the increased number of meals and the increased intakes of many nutritious foods such as; animal food items, fruits and vegetables and dairy foods. The percentage of adolescents having breakfast daily of high and low SES was 74.5% vs 55% in boys and 65.6% vs 45% in girls. The percentage of girls with refugee status who had lunch was higher (90.2%) compared to the local citizen girls (83.9%), (p = 0.03). Girls were less likely to skip daily lunch (OR = 0.55, 95% CI = 0.36–0.87, p = 0.01) compared to boys. Risk of skipping lunch was three times higher among adolescents living in the village compared to Gaza well-off area (OR = 3.3, 95% CI = 1.72–6.31, p < 0.001). Adolescents who were having lunch daily were less likely to skip breakfast or dinner. Only 11.6% of boys and 16.2% of girls consumed fruits daily. In multivariate analysis, SES was positively associated with food frequency intake scores in both genders. Boys from the refugee camp and the village had a significant higher consumption of fruits and vegetables than boys from high and low income area in Gaza City, while it was the opposite in girls.

**Conclusion:**

Meal skipping is common, particularly among those of low SES and the intakes of many nutritious foods such as animal food items, fruits and vegetables and dairy foods seem to be low among adolescents of low SES. The results of this study could be used as an important base-line for future monitoring of the nutritional situation of adolescents.

## Background

It is well known that adolescence is a period of rapid growth and maturation in human development, and that extra nutrients are needed to support their growth spurt. Healthy eating patterns in childhood and adolescence promote optimal childhood health, growth, and intellectual development, as well as prevent health problems later in adulthood and old age [1,2]. Most [3-7] but not all [8] studies show an inverse association between the consumption of healthy foods, such as fruits and vegetables, and risk of cardiovascular disease and all-cause mortality. Adolescent food habits are an important concern in the present accelerated nutrition transition [9]. Food habits established in childhood and adolescence tend to be stable into adulthood [10-12]. Nutritional deficiencies and poor eating habits established during adolescence can have long-term consequences, including delayed sexual maturation and lower final adult height [13]. Nutritional surveys in the US have shown that the highest prevalence of nutritional deficiencies occurs during this period of growth, and more than 90% of adolescents report to be eating snacks between meals, usually processed and high-fat items. These snacks may supply as much as one-third of the total daily nutrient intake.

Diet-related chronic diseases such as coronary heart disease (CHD), hypertension, diabetes and cancer have become the major health problems in most Arab Middle East countries. Several factors have contributed to the high prevalence of these diseases including the rapid change in food consumption patterns and socioeconomic status during the last four decades [14].

The Palestinians have been subject to a number of changes in their lifestyle during the past decades. Some of these are part of larger changes affecting the whole region, such as the undergoing transition in health and lifestyle [15]. Food consumption patterns have changed to a more 'westernized' diet with high intake of foods rich in fat, cholesterol, free sugars and sodium and low in dietary fiber [16]. Other nutritional changes that have occurred are unique to the Palestinian political situation, such as those caused by border closure policy which has affected households' economies and hence their access to and ability to purchase food. In becoming refugees; many Palestinians lost access to their traditional food bases which was characterized by a high-fiber content and was low in fat and cholesterol. In addition, the close attachment to the Israeli economy, the links to the global market, and the flow of donations given to refugees are important elements of these dietary changes. About two-thirds (71.8%) of the Gaza population receive food assistance from the humanitarian community mainly from United Nations Relief and Works Agency (UNRWA) and World Food Program (WFP) [17]. Household purchasing power remains limited, especially in the Gaza Strip where a greater percentage of households have limited employment and their coping strategies are more frequently utilized for food access. The resulting dietary changes have lead to changes in the health situation of the Palestinian people. A Nutritional Assessment of the West Bank and Gaza Strip survey in 2003 indicated that 45.1% of Gaza households are under- or unemployed and thus had limited purchasing capacity and the amount of monthly household income decreased by 53.9% in Gaza Strip [17]. Gazan households have resorted to a number of coping strategies in order to gain food access; such as purchasing food on credit, decreasing the amount of food consumption and the number of daily meals [17]. We have demonstrated earlier that a nutritional public health problem in North Gaza Strip existed in 2003. Our findings revealed that the prevalence of overweight/obesity was 17.9%, stunting was 9.7% and anemia was 49.6% among the adolescents included in the study [18]. However, availability of specific, timely and accurate nutritional data is still a problem in Palestine. To our knowledge, there is very little information about dietary patterns among adolescents in Palestine and how these vary according to sociodemographic characteristics. Only one recent study on food consumption has previously been carried out in Palestine [19]. Therefore, there is a need to obtain more information on this subject to enable the government and other non-governmental agencies to formulate policies and initiate strategies for the well-being of adolescents. The objective of this article is to describe the associations between sociodemographic factors and meal patterns and food intake among Palestinian school adolescents (12–15 year) in North Gaza Strip.

## Methods

### Setting and Study population

The study locations were selected from the northern part of Gaza Strip including Gaza City. In Gaza city, a high income neighbourhood (*well-off area*) and a low income neighbourhood (*not-well-off area*) was selected. The high income area was characterized with a higher housing standard, better infrastructure and lower population density than the low income neighbourhood. The other two locations were Jabalia refugee camp and Jabalia village. The selected communities cover both urban and semi-urban areas, with Jabalia at the semi-urban end of the spectrum. The communities are in close vicinity and can be considered as one continuous community due to the high population density. Gaza Strip had in 2002 a total of 400,462 students attending 481 schools, of which 197 were preparatory schools, 62 of them were located in Gaza city and Gaza North with 47,442 pupils [20]. Girls and boys are separated in different schools.

The design of this study has been previously described [18]. Briefly, it is a cross-sectional study conducted in preparatory schools during the fall 2002. A multistage cluster sampling technique was employed; in Gaza city, three girls' schools and three boys' schools were randomly selected: downtown in the well-off area (Remal) – two governmental schools and two schools of the United Nations Relief and Works Agency for Palestine Refugees (UNRWA), and in the non well-off area of the city-two governmental schools. In the Jabalia area, two girl schools and two boy schools were randomly selected: One girl school and one boy school operated by the Government in Jabalia village, and one UNRWA girl- and one boy school in Jabalia Refugee camp. In each school three classes were randomly selected representing the three different grades (7^th^–9^th^). Thus, students from 30 classes in 10 schools were invited to join the study. The age range of the study population was 12 to 15 years.

A self-administered pre-coded questionnaire was filled in by the students in the classroom within 45 minutes time in the presence of the researcher and was thereafter collected by him. The researcher had beforehand gone through and explained all the questions to the students. In addition, a parent's self-administered questionnaire was used in order to collect the data that students were unable to provide or embarrassed to talk about. These questionnaires were brought home by the students to be filled in by the parents or by other main providers and were returned to the teacher the next day. The participants were informed that answering the questionnaire was anonymous and entirely voluntary and that the information would be treated confidentially. The study was approved by the Ministry of Health and the Helsinki Ethical Committee in Gaza. Ethical clearance was also given by the Regional Medical Research Ethical Committee in Norway. A written consent was obtained from the adolescents' parents and school headmaster. The questionnaire was piloted prior to the survey and adjusted accordingly. Age of all students was obtained by date of birth of the students from the school records.

### Sociodemographic factors

**Resident status **was divided in two groups according to resident status in the sample; refugees and local citizens.

**Household socioeconomic status (SES) **score was constructed based on the possession of household amenities. Ownership of a car, a video or a DVD, a TV, a satellite antenna, a computer and a refrigerator were each given a value of 1 and summed. The SES score was divided in three categories: *low*: 2 or less; *medium*: 3 to 4, and *high*: 5 to 6.

**Residential area **was grouped into four categories based on the location of the schools: Gaza City *well-off and non well-off, village *and *refugee camp*.

**Age of all students **was obtained from date of birth in their school records. Four age groups were constructed: age group 12: 11.5 – <12.5 years, age group 13: 12.5 – <13.5 years, age group 14: 13.5 – <14.5 years; and age group 15: 14.5 – <15.5 years.

**Father's job status **was based on current employment status and was dichotomized into *unemployed *and *employed*.

**Father's educational level **was divided into three categories based on the number of years of education; *low*: 11 years or less, *medium*: 12 to 14 years, and *high*: 15 years and more.

**Mother's educational level **was constructed in the same way as fathers, but due to very few subjects in the highest educational level, the two last categories were collapsed to one, i.e. *medium/high*.

### Food and meals frequency questionnaire

#### Food frequency questionnaire(FFQ)

A food frequency questionnaire including 42 food items was developed, in which 25 of the items were taken from a questionnaire developed by Stene *et al *for the West Bank representing 40% of the energy intake at household level [15]. The other items were taken from Whitney et al [21] and adopted to the dietary situation in Gaza Strip. Portion sizes were not included in the questionnaire. The students were asked "How many times per week or per day do you eat the following food items? The answer categories ranged from 0 to 7 times (8 categories), either per day or per week. The subjects were instructed to exclude tomatoes and cucumbers when they answered the question on consumption of vegetables, since these are traditionally served at each meal and has been affordable to all SES groups in Gaza at dumping prices. For data analysis, the food items were collapsed into sixteen food groups. The meal pattern question was "How many of these meals (breakfast, lunch, dinner and snacks) have you eaten during the last week?". The distribution of the number of meals per week suggested a normal pattern of 7 meals per week, and a "deviant" pattern of less than 7 meals per week. Hence these variables were dichotomized: 7 times per week or less than 7 times per week for all meals. The frequency intakes of the different food items were dichotomized according to their natural distribution, and were thus dichotomized into < 7 times per week as the common pattern and ≥ 7 times per week (deviant pattern). Meat and chicken were consumed less often and therefore grouped into less than 3 times per week as the common pattern and more and equal 3 times per week as the deviant pattern. While bread and tea were consumed almost three times daily; therefore, they were grouped less than 20 times per week (common pattern) or into equal or more than 20 times per week (deviant pattern). Prevalences of the deviant patterns are reported in the results.

#### Food frequency intake score

Six food frequency scores were constructed based on selected food items from the FFQ. They were constructed by the sum of recoded responses (times/week) for the food items included in each score. The scores included the following items: 1)*Animal foods*: beef, turkey/chicken, liver, fish, hamburger, shawerma/kebab, canned meat and eggs. 2) *Fruits and vegetables (FV foods)*: fruits, fruit juice, salad and fresh and cooked vegetables (Tomatoes and cucumbers not included). 3) *Milk foods*: yogurt, white cheese, yellow cheese and *lebneh *(yogurt cheese). 4) *Sweet foods*: honey, jam, jelly, cookies, *kunafeh *(Arabic cheese cake), chocolates, biscuits and soft drinks. 5) *Western foods*: hamburger, canned meat, macaroni, yellow cheese, soft drinks, pizza and potato chips. And 6) *Traditional foods*: lentils, *hommos *(chick pea paste), *falafel *(deep fried chick pea balls) and fava beans.

### Statistical analysis

The Statistical Package for Social sciences (SPSS for windows, version 14.0) was used to analyze the data. Statistical significance was considered at p < 0.05. Chi square test was used to compare differences between boys and girls. The food patterns were analyzed both at continuous (number of meals per week) and categorical (percentage exceeding a given number of meals per week). The meal patterns were only analyzed as categorical numbers. For the continuous outcomes we presented mean numbers of meal per week, we tested the differences between groups with T-tests or ANOVA, and we did linear regression analyses. For the categorical outcomes, we presented percentage exceeding a given number of meals per week, we tested the differences between groups with chi-square tests, and logistic regression analyzes. Lunch, which is the main meal among Palestinian community, was used as outcomes in logistic regression models. In the linear regression on three food scores (animal foods score, F/V foods score and Milk foods score) we tested interactions between sex and resident status, and between sex and residential area. We found several significant interactions, in particular, that the effects of residential area on all three food scores were significantly different for boys and girls. We, therefore, presented separate tables for boys and girls for these outcomes.

## Results

### Description of the sample

Among the 1221 invited students, 944 were recruited (response rate: 77.3%). Of the 277 non-respondents; 25 withdrew, 63 were absent on the day of the interview, 70 parents refused on behalf of their children (no students refused on own behalf), 105 did not complete the questionnaire and 14 were falling outside the age brackets 11.5–15.5 years. The sample had a higher proportion of girls (53.5%) than boys, and a higher proportion of pupils with refugee status (60.4%) than local citizens. About 40% of the pupils lived in the well-off area and 22.2% in the non-well-off area of Gaza city, while 15.5% of the sample lived in Jabalia village and 22.1% lived in Jabalia refugee camp. About one-third of the sample lived in crowded dwellings (>3 persons/room) and nearly half of the pupils' fathers were unemployed. One-fourth of the sample was from families with the lowest socioeconomic status and another one-fourth came from households of high socioeconomic status. There were more fathers with higher education than mothers (Table 1).

**Table 1:** Sociodemographic characteristics of the study population

***Sociodemographic Factors***	***N*** ***944***	***%***
**Gender**		
Boys	439	46.5
Girls	505	53.5
**Resident status**		
Local citizen	372	39.6
Refugee	568	60.4
**Residential area**		
Gaza (well-off)	379	40.1
Gaza (non-well-off)	210	22.2
Village	146	15.5
Camp	209	22.1
**Age in years**		
12 yrs	161	17.1
13 yrs	295	31.3
14 yrs	312	33.1
15 yrs	176	18.6
**Crowding Index**		
≤ 3 persons/room	610	71.8
> 3 persons/room	240	28.2
**Father's working Status^*a*^**		
Unemployed	430	46.2
Employed	501	53.8
**Household SES^*b*^**		
low	245	26.7
medium	439	47.8
high	234	25.5
**Father's education^*c*^**		
< 12 years	486	52.8
12 – 14 years	262	28.4
> 14 years	173	18.8
**Mother's education^*d*^**		
< 12 years	532	57.6
> 12 years	391	42.4

^*a *^Father's job status was based on the current employment status.

^*b *^Household SES score was based on the possession of 6 household amenities; each item was given a value of 1.

^*c*^Father's education level was divided into three categories based on the numbers of years of education; (1) low was considered 11 years or less, (2) medium was considered 12 to 14 years, and high was considered 15 years and more.

^*d *^Mother's education level was divided into two categories based on the numbers of years of education; (1) low was considered 11 years or less, (2) medium/high was considered 12 or more.

### Meal Patterns

Tables 2 (boys) and 3 (girls) show that lunch was the meal eaten most often; more than 80% of the adolescents had lunch daily, but the proportion was higher in girls than boys (87.7% vs 82.5; p < 0.05). Dinner was eaten less often, with only half of the sample having this meal daily. Breakfast was eaten daily by 62% of the adolescents, with a higher proportion of boys than girls having this meal daily (65.6% vs 59% respectively, p < 0.05). In the total sample, less than 40% had all three meals per day with a higher proportion among boys than girls (41.9% vs 36.4%, p < 0.05). There was a trend of a higher percentage having daily meals with increasing SES. Significant associations (p < 0.05) were found between SES and for each of the three meals in girls, while among boys significance was found only for breakfast and lunch (Tables 2 and 3). The difference in meal pattern between adolescents with refugee status and those with local citizen status was small. The difference was only significant for girls in regard to lunch (83.9% among refugees vs 90.2% among local citizens, p = 0.03). Also mother's education had little effect on proportions having daily meals. Only in regard to lunch among girls', mother's education seemed to have a significant positive effect on the proportion having lunch daily (p = 0.005). Daily intake of snacks did not show any difference across socioeconomic status.

**Table 2:** Meal patterns and food intake of Palestinian adolescent boys (n = 439) from North Gaza Strip by sociodemographic factors (%)^*a*^

		**Resident status^*b*^**			**Household SES^*c*^**				**Mother education^*f*^**		
				
	**Total** **(%)**	**Local Citizen** **(n = 186)**	**Refugee** **(n = 253)**	**P**	**Low** **(n = 131)**	**Medium** **(n = 190)**	**high** **(n = 106)**	**p**	**Low** **(n = 242)**	**Medium** **/high** **(n = 184)**	**p**
**Meal pattern (7 t/w)^*d*^**											
Breakfast	65.6^†^	64.0	66.8	0.30	55.0	67.9	74.5	0.005	66.1	65.8	0.51
Lunch	82.5^†^	81.7	83.0	0.41	75.6	84.7	86.8	0.04	78.1	88.0	0.005
Dinner	49.5	47.8	50.6	0.32	45.8	51.1	50.9	0.61	51.7	46.2	0.15
3 meals/day	41.9^†^	37.6	45.6	0.07	34.4	42.6	49.6	0.07	51.7	46.2	0.15
Snacks	25.1	26.3	24.1	0.37	25.2	21.6	28.3	0.42	24.8	25.5	0.47
**Food intake (weekly basis)^*e*^**											
Beef (>3 t/w)	7.3	7.0	7.5	0.41	6.1	6.8	10.4	0.42	6.2	8.2	0.28
Chicken (>3 t/w)	3.4	1.1	5.1	0.02	2.3	1.6	8.5	0.005	2.1	5.4	0.05
Bread (≥ 20 t/w)	76.1^†^	74.7	77.1	0.32	65.6	80.5	81.1	0.003	73.1	79.9	0.07
Cookies (≥ 7 t/w)	14.6^†^	9.1	18.6	0.004	13.0	13.7	18.9	0.38	15.7	12.0	0.17
Soft drinks (≥ 7 t/w)	6.2^†^	1.6	9.5	<0.001	5.3	5.8	7.5	0.76	5.8	7.1	0.37
Eggs (≥ 7 t/w)	13.9	5.9	19.8	<0.001	15.3	11.6	17.0	0.39	11.6	16.3	0.10
Rice (≥ 7 t/w)	6.8^†^	2.7	9.9	0.002	6.1	6.3	8.5	0.72	5.8	8.2	0.22
Fruits (≥ 7 t/w)	11.6^†^	9.1	13.4	0.11	6.9	11.1	17.0	0.049	11.6	11.4	0.54
Juice (≥ 7 t/w)	7.5^†^	3.8	10.3	0.005	4.6	6.8	13.1	0.04	6.6	9.2	0.20
Chocolates (≥ 7 t/w)	10.7^†^	5.4	14.6	0.001	7.6	10.5	16.0	0.12	11.6	10.3	0.40
Yogurt (≥ 7 t/w)	19.8	16.1	22.5	0.006	19.1	17.9	20.8	0.83	17.8	21.7	0.18
Vegetables (≥ 7 t/w)	27.6^†^	21.0	32.4	<0.001	25.2	30.0	27.4	0.63	28.1	27.1	0.51
Milk (≥ 7 t/w)	33.7	31.2	35.5	0.19	31.3	31.1	39.6	0.28	34.7	33.2	0.41
Cheese (≥ 7 t/w)	30.8	24.7	35.2	0.02	16.8	31.6	47.2	<0.001	27.3	33.7	0.09
Legumes (≥ 7 t/w)	18.9^†^	17.7	19.8	0.40	16.8	19.5	21.7	0.63	19.0	18.5	0.50
Tea (≥ 20 t/w)	32.8	30.6	34.4	0.25	31.3	35.3	30.2	0.61	36.4	27.7	0.04

^†^χ^2 ^test was used to compare differences between boys and girls. Statistical significance was considered at, p < 0.05

^*a *^χ^2 ^was used to compare differences between sociodemographic variables. P < 0.05 considered to be significant

^*b *^Palestinian population is divided into two Resident status groups; refugees and local citizen of which 70% of the Gaza Population is with refugee status while 30% of them are with local citizen status.

^*c *^Household SES score was based on the possession of 6 household amenities; each item was given a value of 1.

^*d*^There are three meals consumed on a daily basis in the Palestinian context, in which lunch is the main family meal.

^*e*^Foods items frequency intake was calculated on weekly basis and grouped into two categories according to their weekly consumption. Meat and chicken were grouped as > 3 times and 3 times or less per week, bread was grouped as < 20 times and ≥ 20 times per week and all other food items were grouped as <7 times and ≥ 7 times per week.

^*f *^Mother's education level was divided into two categories based on the numbers of years of education; (1) low was considered 11 years or less, (2) medium/high was considered 12 or more.

**Table 3:** Meal patterns and food intake of Palestinian adolescent girls (n = 505) from North Gaza Strip by sociodemographic factors (%)^*a*^

		**Resident status^*b*^**			**Household SES^*c*^**				**Mother education^*f*^**		
				
	**Total** **(%)**	**Local** **Citizen** **(n = 186)**	**Refugee** **(n = 315)**	**P**	**Low** **(n = 114)**	**Medium** **(n = 249)**	**High** **(n = 128)**	**p**	**Low** **(n = 290)**	**Medium** **/high** **(n = 207)**	**p**
**Meal pattern (7 t/w)^*d*^**											
Breakfast	59.0^†^	57.0	60.3	0.26	45.6	61.4	65.6	0.003	57.2	59.9	0.31
Lunch	87.7^†^	83.9	90.2	0.03	83.9	90.4	89.8	0.02	85.9	89.9	0.12
Dinner	46.5	45.2	47.6	0.33	36.0	47.0	54.7	0.01	47.6	45.4	0.35
3 meals/day	36.4^†^	32.8	38.7	0.11	21.1	38.6	46.1	<0.001	47.6	45.4	0.35
Snacks	21.1	18.8	22.2	0.21	14.0	22.5	20.8	0.13	20.7	20.8	0.53
**Food intake (weekly basis)^*e*^**											
Beef (>3 t/w)	7.9	8.6	7.6	0.49	2.6	7.6	7.9	0.01	6.6	9.7	0.14
Chicken (>3 t/w)	3.8	3.2	4.1	0.40	0.9	3.6	7.0	0.04	3.8	3.9	0.57
Bread (≥ 20 t/w)	68.9^†^	66.7	70.5	0.21	66.7	72.3	65.6	0.33	70.0	66.7	0.24
Cookies (≥ 7 t/w)	19.6^†^	10.8	25.1	<0.001	14.0	18.1	28.1	0.01	18.3	21.3	0.24
Soft drinks (≥ 7 t/w)	9.3^†^	4.3	12.4	0.002	5.3	9.6	13.3	0.11	7.6	10.6	0.15
Eggs (≥ 7 t/w)	14.3	5.9	19.0	<0.001	6.1	15.7	20.3	0.01	12.7	17.4	0.08
Rice (≥ 7 t/w)	10.1^†^	2.7	14.3	<0.001	2.6	14.5	9.4	0.003	7.9	13.5	0.03
Fruits (≥ 7 t/w)	16.2^†^	11.3	19.4	0.01	8.8	16.1	21.9	0.02	14.5	18.4	0.15
Juice (≥ 7 t/w)	14.7^†^	9.1	17.8	0.007	8.8	14.1	21.9	0.01	14.8	14.0	0.45
Chocolates (≥ 7 t/w)	17.0^†^	10.8	21.0	0.002	8.8	17.7	24.2	0.01	15.9	17.9	0.32
Yogurt (≥ 7 t/w)	20.8	21.0	20.6	0.51	14.9	19.7	29.7	0.01	20.0	22.2	0.31
Vegetables (≥ 7 t/w)	34.1^†^	18.8	42.9	0.005	20.2	32.1	52.3	<0.001	30.0	39.1	0.02
Milk (≥ 7 t/w)	29.9	29.6	30.5	0.47	17.5	30.9	38.3	0.002	26.9	34.3	0.047
Cheese (≥ 7 t/w)	35.0	26.3	40.6	0.001	15.8	34.1	54.7	<0.001	26.6	46.9	<0.001
Legumes (≥ 7 t/w)	14.7^†^	14.0	15.2	0.34	13.2	16.1	14.1	0.74	15.5	14.0	0.37
Tea (≥ 20 t/w)	29.9	29.6	30.2	0.49	31.6	29.3	28.1	0.84	31.7	26.6	0.13

^†^χ^2 ^test was used to compare differences between boys and girls. Statistical significance was considered at, p < 0.05

^*a *^χ^2 ^was used to compare differences between sociodemographic variables. P < 0.05 considered to be significant

^*b *^Palestinian population is divided into two Resident status groups; refugees and local citizens. In which 70% of the Gaza Population is with refugee status while 30% of them are with local citizen status.

^*c *^Household SES score was based on the possession of 6 household amenities; each item was given a value of 1.

^*d*^There are three meals consumed on a daily basis in the Palestinian context, in which lunch is the main family meal.

^*e*^Foods items frequency intake was calculated on weekly basis and grouped into two categories according to their weekly consumption. Meat and chicken were grouped as > 3 times and 3 times or less per week, bread was grouped as < 20 times and ≥ 20 times per week and all other food items were grouped as <7 times and ≥ 7 times per week.

^*f *^Mother's education level was divided into two categories based on the numbers of years of education; (1) low was considered 11 years or less, (2) medium/high was considered 12 or more.

Results from multivariate analysis showed that girls were less likely to skip daily lunch (OR = 0.55, 95% CI = 0.36–0.87, p = 0.01) compared to boys. The risk of skipping lunch was three times higher among adolescents living in the village compared to Gaza well-off area (OR = 3.3, 95% CI = 1.72–6.31, p < 0.001). Also, adolescents with mothers of medium/high level education had lower risk of skipping lunch compared to those with mothers of low level of education (OR = 0.59, 95% CI = 0.36–0.99, p = 0.047). The analysis also revealed that those who were eating breakfast or dinner daily were less likely to skip lunch. The significant association between household SES and lunch skipping disappeared in the multivariate analysis model (Table 4).

**Table 4:** Multivariate logistic regression of lunch meal pattern of Palestinian adolescents from North Gaza Strip by Sociodemographic factors (n = 944)^*a*^

	**Lunch ≤ 6 time/week^*b*^**			**Multivariate logistic regression**	
		
	**N**	**%**	**p**	**OR (95% CI**)	**p**
**Gender**					
Boys (n = 439)	77	17.5	0.015	**1**	
Girls (n = 505)	62	12.3		**0.55 (0.36–0.87)**	**0.01**
**Resident status**					
Local citizen (n = 372)	64	17.2	0.048	1	
Refugee (n = 568)	74	13.0		0.82 (0.46–1.48)	0.51
**Residential area**					
Gaza (well-off) (n = 379)	47	12.4	<0.001	**1**	
Gaza (non-well-off) (n = 210)	19	9.0		0.46 (0.21–1.05)	0.06
Village (n = 146)	45	30.8		**3.30 (1.72–6.31)**	**<0.001**
Camp (n = 209)	28	13.4		1.21 (0.65–2.23)	0.55
**Age in years**					
12 yrs (n = 161)	18	11.2	0.24	1	
13 yrs (n = 295)	45	15.3		1.52 (0.56–3.13)	0.26
14 yrs (n = 312)	43	13.8		1.17 (0.56–2.45)	0.67
15 yrs (n = 176)	33	18.8		1.72 (0.79–3.71)	0.17
**Crowding Index**					
≤ 3 persons/room (n = 610)	73	12.0	0.001	1	
> 3 persons/room (n = 240)	49	20.4		1.37 (0.83–2.27)	0.22
**Father's working Status**					
Unemployed (n = 501)	77	17.9	0.009	1	
Employed (n = 430)	61	12.2		0.92 (0.55–1.54)	0.76
**Household SES**					
Low (n = 245)	54	22.0	0.001	1	
Medium (n = 439)	53	12.1		0.73 (0.42–1.29)	0.28
High (n = 430)	27	11.5		1.17 (0.58–2.38)	0.66
**Father's education**					
Low (n = 486)	88	18.1	0.013	1	
Medium (n = 262)	31	11.8		0.70 (0.40–1.21)	0.20
High (n = 173)	18	10.4		0.93 (0.47–1.68)	0.85
**Mother's education**					
Low (n = 532)	94	17.7	0.005	**1**	
Medium/High (n = 391)	43	11.0		**0.59 (0.36–0.99)**	**0.047**
**Breakfast (n = 944)**					
≤6 t/w (n = 358)	100	27.9	<0.001	**1**	
= 7 t/w (n = 586)	39	6.7		**0.21 (0.13–0.34)**	**<0.001**
**Dinner**					
≤6 t/w (n = 492)	113	23.0	<0.001	**1**	
= 7 t/w (n = 452)	26	5.8		**0.34 (0.20–0.59)**	**<0.001**
**Snacks**					
≤6 t/w (n = 728)	121	16.6	0.001	1	
= 7 t/w (n = 216)	18	8.3		0.75 (0.39–1.45)	0.39

^*a *^Multivariate logistic regression dependant variable is lunch ≤ 6 time/week, independent variables are all sociodemographic

variables, and breakfast, dinner and snacks.

^*b*^χ^2 ^was used to compare differences between dependant variable is lunch ≤ 6 time/week and independent variables are all

sociodemographic variables, and breakfast, dinner and snacks. P < 0.05 considered to be significant.

### Food frequency intake

The study revealed that more girls than boys had a higher frequency intake of fruits, juice, vegetables, rice and sugary food items such as soft drinks, chocolates and cookies. There were no significant differences between the genders in the percentages consuming milk, yogurt, meat and chicken above the given frequency cut-offs. However, boys were found to consume bread more often than girls (Tables 2 and 3).

A higher percentage of adolescents with refugee status tended to eat more frequently most of the food items listed in tables 2 and 3 compared to those with local citizenship. The consumption of meat was generally low in this study. However, the proportion consuming beef and chicken >3 t/w increased according to SES. Only 11.6% of boys and 16.2% of girls had fruits daily. The figures were low in all SES groups, but increased with higher SES. A similar picture was found for vegetable intake other than tomatoes and cucumbers. According to the data, less than 30% of students had such vegetables daily. A high proportion of students reported daily intake of sweetened tea in all SES groups; with no significant change in proportions across sociodemographic groups (Tables 2 and 3). The tables revealed significant differences between groups with unequal levels of mother's education, but only among girls; rice, vegetables, milk and cheese were the items consumed more often by those with mothers of higher education.

When frequency consumption of the different food items were aggregated to six different scores and analyzed according to sociodemographic factors, significant differences were found for most of the scores. Differences between genders were found in fruits/vegetable score and western food score; girls tended to eat more often from these two food groups. Adolescents with refugee status and those living in the well-off part of Gaza city had a significant higher mean frequency intake of all six food groups than those living in the non-well off area of Gaza city. Adolescents living in less crowded families, with employed fathers, with high SES and with high parents' level of education showed also a higher frequency of consumption of animal foods, milk products, fruits/vegetables, sweet and western food than other groups (Table 5). Residence was the only variable associated with consumption of traditional foods score; adolescents from Jabalia refugee camp having the highest score. The multivariate linear regression analysis revealed that residential area was significantly associated with animal, F/V and milk food scores in boys and girls (Table 6). Boys from the refugee camp and the village had a significantly higher consumption of F/V than boys from the well-off area of Gaza City, while it was the opposite in girls. Girls from the non-well-off area, village and refugee camp had significant lower consumption of milk foods than the well-off area, while this difference was significant in boys from the village only. Household SES was positively associated with the same three food scores in both boys and girls. Resident status in boys and father's work status in girls were associated with these three food scores. In girls, intake of FV increased significantly with the educational level of father (p = 0.04), while such associations were not found in boys.

**Table 5:** Mean food score times per week by sociodemographic factors among Palestinian adolescents in North Gaza Strip (n = 944)

**Sociodemographic factor**	**Animal foods^a^**		**Milk foods^b^**		**F/V foods^c^**		**Sweet foods^d^**		**Western foods^e^**		**Traditional foods^f^**	
	
	**Mean**	**p**	**Mean**	**p**	**Mean**	**p**	**Mean**	**p**	**Mean**	**p**	**Mean**	**p**
**Gender (n = 944)**												
Boys	7.29	0.10	10.19	0.33	7.99	0.02	10.07	0.13	12.13	0.02	9.03	0.11
Girls	6.65		11.03		9.03		11.04		13.88		7.97	
**Resident status (n = 940)**												
Local citizen	5.61	<0.001	8.64	<0.001	6.70	0.001	8.35	<0.001	9.46	<0.001	7.63	0.04
Refugee	7.84		11.97		9.74		12.08		15.47		9.06	
**Residential area (n = 944)**												
Gaza (well-off)	8.07	<0.001	14.46	<0.001	9.66	0.001	12.70	<0.001	17.44	<0.001	8.39	0.01
Gaza (non-well-off)	4.96		7.87		6.25		7.40		8.08		7.26	
Village	6.47		8.25		7.32		8.30		9.94		7.66	
Camp	7.25		8.17		9.66		11.58		12.33		10.36	
**Age in years**												
12 yrs	7.32	0.52	11.65	0.54	7.81	0.004	11.21	0.45	14.22	0.50	9.00	0.71
13 yrs	6.54		11.03		7.69		10.02		12.51		8.59	
14 yrs	7.13		9.98		9.44		10.41		12.98		8.46	
15 yrs	6.98		10.22		9.01		11.30		13.08		7.74	
**Crowding Index (n = 835)**												
≤ 3 persons/room	7.56	<0.001	12.01	<0.001	9.10	0.001	11.48	<0.001	14.61	<0.001	8.69	0.39
>3 persons/room	5.68		7.58		7.47		8.39		9.79		8.01	
**Father's working Status (n = 931)**												
Unemployed	5.65	<0.001	8.11	<0.001	7.50	<0.001	9.13	<0.001	10.22	<0.001	8.36	0.68
Employed	8.09		12.80		9.42		11.89		15.47		8.63	
**Household SES (n = 918)**												
Low	5.44	<0.001	6.17	<0.001	6.82	<0.001	7.60	<0.001	8.63	<0.001	7.93	0.54
medium	6.81		10.35		8.57		10.63		13.12		8.68	
High	9.03		15.88		10.40		13.85		18.18		8.91	
**Father's education (n = 921)**												
Low	5.81	<0.001	9.33	<0.001	7.44	<0.001	9.29	<0.001	11.15	<0.001	8.23	0.50
Medium	8.39		11.97		9.71		12.29		14.84		9.14	
High	8.01		11.98		9.90		11.65		15.43		8.33	
**Mother's education (n = 923)**												
Low	6.22	<0.001	9.25	<0.001	7.99	0.003	10.03	0.048	11.47	<0.001	8.72	0.42
Medium/High	7.97		12.35		9.28		11.33		15.10		8.18	

^a ^Animal foods includes weekly consumptions of beef, turkey/chicken, liver, fish, hamburger, shawerma/kebab, canned meat and eggs.

^b ^Milk foods includes weekly consumptions of milk, yogurt, white cheese, yellow cheese and lebneh.

^c ^Fruits and vegetables includes weekly consumptions of fruits, fruits juice, salad and fresh and cooked vegetable (tomatoes and cucumbers are not included).

^d ^Sweet foods includes weekly consumptions of honey, jam, jelly, cookies, kunafeh, chocolates, biscuits and soft drinks.

^e ^Western foods includes weekly consumptions of hamburger, canned meat, macaroni, yellow cheese, soft drinks, pizza and potato chips.

^f ^Traditional foods includes weekly consumptions of lintels, hommos, falafel and fava beans.

ONE WAY ANOVA was used to compare difference among groups.

**Table 6:** Multiple linear regression of the relationship between food score and sociodemographic factors among Palestinian school adolescents in North Gaza Strip by gender (n = 944)

	**Boys(n = 439)**						**Girls(n = 505)**					
	
**Sociodemographic Factors**	**Animal foods^a^**		**F/V foods^b^**		**Milk foods^c^**		**Animal foods^a^**		**F/V foods^b^**		**Milk foods^c^**	
	
	***B***	***p***	***B***	***p***	***B***	***p***	***B***	***p***	***B***	***P***	***B***	***p***
**Constant**	4.97		2.69		7.92		6.60		8.15		12.90	
**Resident status**												
Local citizen	Ref.		Ref.		Ref.		Ref.		Ref.		Ref.	
Refugee	2.06	0.01	2.48	<0.001	3.95	0.03	-0.07	0.93	0.33	0.70	-3.06	0.10
**Residential area**												
Gaza (well-off)	Ref.		Ref.		Ref.		Ref.		Ref.		Ref.	
Gaza (non-well-off)	0.68	0.46	1.61	0.12	-0.84	0.70	-3.25	<0.001	-4.53	<0.001	-7.43	<0.001
Village	0.79	0.28	2.65	<0.001	-5.37	<0.001	-1.54	0.04	-1.30	0.09	-4.33	0.01
Camp	1.92	0.03	4.49	<0.001	-1.76	0.38	-2.30	0.01	-6.05	<0.001	-5.95	<0.001
**Age in years**												
12 yrs	Ref.		Ref.		Ref.		Ref.		Ref.		Ref.	
13 yrs	-0.67	0.42	0.78	0.40	-0.10	0.96	0.21	0.79	0.35	0.67	0.34	0.84
14 yrs	0.09	0.92	2.18	0.02	-1.24	0.52	1.07	0.18	3.16	<0.001	0.80	0.64
15 yrs	-0.28	0.77	1.58	0.13	-1.21	0.57	1.33	0.14	2.96	<0.001	0.65	0.74
**Father work status**												
Employed	Ref.		Ref.		Ref.		Ref.		Ref.		Ref.	
Unemployed	-0.77	0.23	-0.75	0.29	-0.33	0.82	-2.15	<0.001	-1.26	0.05	-3.02	0.02
**Household SES**												
Low	Ref.		Ref.		Ref.		Ref.		Ref.		Ref.	
Medium	-0.05	0.94	0.72	0.34	2.13	0.17	1.42	0.05	1.22	0.10	3.63	0.02
High	1.38	0.09	1.68	0.07	6.64	<0.001	2.52	0.01	2.30	0.01	6.46	<0.001
**Fathers level of education**												
Low	Ref.		Ref.		Ref.		Ref.		Ref.		Ref.	
Medium	1.07	0.12	1.39	0.07	-0.77	0.63	1.65	0.01	0.91	0.18	0.99	0.49
High	0.42	0.62	0.76	0.41	-3.20	0.10	0.26	0.76	1.76	0.04	-0.07	0.97
**Mothers level of education**												
Low	Ref.		Ref.		Ref.		Ref.		Ref.		Ref.	
Medium/high	0.96	0.11	0.05	0.94	1.48	0.29	-0.01	0.99	-0.27	0.68	1.47	0.28

^a ^Animal foods includes weekly consumptions of beef, turkey/chicken, liver, fish, hamburger, shawerma/kebab, canned meat and eggs.

^b ^F/V foods includes weekly consumptions of fruits, fruits juice, salad and fresh and cooked vegetable (tomatoes and cucumbers are not included).

^c ^Milk foods includes weekly consumptions of milk, yogurt, white cheese, yellow cheese and lebneh.

## Discussion

### Main findings

The study showed that meal skipping was common among school adolescents in Gaza Strip, especially among those with low SES. Dinner was the meal most commonly skipped, and only around 40% consumed all the three daily meals. There were great variations in frequency intake of many foods according to sociodemographic variables. Boys tended to eat more often bread and legumes, while girls consumed more often fruits and vegetables and western foods. The difference in consumption pattern was especially pronounced when comparing boys and girls of different socioeconomic status; the higher the status the more often most of the listed food items were consumed. Interestingly, adolescents with refugee status consumed more often most of the food items listed than those with local citizen status. This difference was however only significant for boys in the multiple linear regression.

### Meal pattern

It is considered important that adolescents have regular daily meals [22] with special attention to breakfast, which is often referred to as the most important meal of the day [23]. However, breakfast skipping is a worldwide problem among adolescents [24,25] and our result is consistent with many studies from the western countries [24,26,27].

The usual number of daily meals in the Palestinian context is three; breakfast, lunch and dinner. Lunch is considered the main meal, and is shared by family members in the home. The findings showed that breakfast and dinner were the meals most often skipped; 59% of the girls and 66% of the boys had breakfast daily. The corresponding figures for dinner were even less; 46.5% and 49.5%, respectively. The prevalence of meal skipping, particularly breakfast, was found by Al Sabbah et al in 2004 to be lower in West Bank than in Gaza [19]. The results on breakfast skipping from the present study cannot be compared with those of Al Sabbah et al's, due to the use of different cut-offs in determining prevalences, but the percentages found are much higher than in some industrialized countries [25,28]. Adolescents from Australia reported lack of time and not being hungry in the morning as the two most common reasons for skipping breakfast [25]. Meal skipping has been shown to be associated with a higher snacking frequency among adolescents [28,29]. When lunch or dinner is missed they are frequently replaced by snacks. Findings from Saudi Arabia showed that breakfast eaters generally consumed more daily calories [30]. However Resnicow found that breakfast skipping was not replaced by snack consumption among US school children aged 9–19 years old [31]. The present study revealed that skipping of lunch was positively associated with breakfast and dinner skipping, and that there was no association between number of meals and snacking frequency. This implies that adolescents who frequently skipped one meal were more likely to skip other meals, thus snacking did not seem to make up for skipped meals. We also found that number of meals was positively associated with SES. Thus poverty could be the main reasons of adolescents skipping breakfast and dinner.

### Food consumption patterns

The results of this study revealed that the food frequency consumption patterns of adolescents in Gaza Strip depended on socio-economic status. The poor consumed the foods included in the frequency list less often. Al Sabah et al [19] found in a similar study from 2004 that adolescents in the Gaza Strip consumed less frequently fruit, meat, chicken, sweets and soft drinks, but more frequently vegetables than their counterparts in the West Bank. Calculating the 95% Confidence interval and comparing the results of our study with the results of Al Sabah et al on adolescents in the Gaza Strip [19]; we found significant differences in proportion of adolescents consuming the above mentioned food items daily: A higher proportion consumed milk in 2002 compared to findings in 2004, otherwise lower proportions consumed fruit, soft drinks, sweets in 2002. There was no significant difference in meat and chicken consumption. The proportion consuming vegetables daily was much lower than what was found by Al Sabbah two years later (1/3 vs 1/2 of the study samples of 2002 and 2004). However this difference may be mostly due to the fact that tomatoes and cucumbers were excluded from the food frequency question on vegetables in the present study.

In addition, this study demonstrated differences in food consumption patterns between boys and girls. Reasons could be differences in gender preferences and cultural norms.

Girls often stay at home, due to cultural norms, where they have easy access to the available foods, such as; fruits, vegetables and sweets. The fact that the girls in the study ate more frequently such foods than boys is consistent with other studies [19]. This may partly explain the relatively high prevalence of overweight girls in Gaza Strip in the same sample [18].

The fact that most foods were eaten more frequently by a higher proportion of adolescents with refugee status compared to those with local citizen status could be due to a higher access to food donations for those with refugee status through United Nation for Palestinian Refugees and Work Agency (UNRWA) and other humanitarian aids agencies.

One important concern regarding food consumption patterns of adolescents is the intake of fruits, fresh juice and vegetables. Fruit consumption was especially low in this study. We did not ask about the consumption of tomatoes and cucumbers, since it has been affordable to all in Gaza at dumping prices, due to lack of export opportunities as a consequence of frequent border closures. Our findings study revealed that the mean frequency of consumption of fruits and those vegetables registered was 8.5 per week. Here, we would assume that adolescents consumed two or three servings per day of tomatoes and cucumbers and very seldom other types of vegetables-Still, we would conclude that the majority of adolescents did not meet the dietary recommendations of WHO on five portions of fruits and vegetables per day [32,33] and the recommendation of eating a variety of different vegetables and fruit [34]. Despite the closures of Gaza borders to the external world and lack of purchasing power which create food inaccessibility [17], some fruits and vegetables, apart from tomatoes and cucumbers, are usually available in the local market and relatively cheap, such as; oranges and strawberries. Thus it could be a promising aspect to launch a campaign on encouraging the community to consume more fruits and vegetables on a daily bases.

The consumption of sugary food items such as soft drinks and cookies was higher among the high SES groups. The prevalence of overweight was higher in the same group as described earlier [18]. Further research is needed to study the association between food intake and nutritional status of school adolescents in Gaza.

The low portion of adolescents in low SES groups that had meat more than three times a week, could possibly be one of the factors contributing to the relatively high prevalence of stunting in the low SES groups, as reported in earlier [18] and in other studies [17,35]. Fish was consumed so infrequently (mean was 0.6 times/week) that it was taken out of the results. Although Gaza Strip is well-known with its good sea food, little of such food has been available in the local market since 1994, due to restrictions imposed on Gaza fishermen by the Israelis. Insufficient consumption of foods rich in protein, vitamins, and minerals is associated with unfavorable overall school performance [36].

Milk and dairy foods seem not to be commonly consumed on a daily bases in this study. The consumption was strongly related to SES, the low SES group consumed milk and dairy foods less than half as often as the high SES group. Several studies have shown a relationship between growth spurt and the risk of fractures [37]. Children and adolescents are recommended to consume food which is high in calcium in order to develop a maximal peak bone mass [38]. The high frequency intake of heavily sweetened black tea is also of concern, particularly among girls because of their higher demands of iron due to menstruation. In Palestine, sweetened black tea is usually consumed immediately after each meal. It is well documented that non-heme iron absorption from foods is inhibited owing to simultaneous tea consumption. A high consumption of tea may thus increase the risk of iron depletion in individuals with marginal iron status [39].

The low consumption of meat, fish and dairy foods, and high intake of tea should be seen in relation to micronutrient deficiencies in general, which are reported to be one of the main public health problems in Palestine. Anemia is affecting more than one-third of Palestinians in general [17,35]. We have earlier reported that anemia was affecting about half of adolescents in the sample from Gaza [18]. About 15% of Palestinian school-age children from 8–10 years old had grade 1 and 2 goitre and 22% of reported cases with Rickets have vitamins A and D deficiency [40].

## Limitations

The study has certain limitations, such as being cross-sectional and the sample being relatively small. The results are not representative of all adolescents in Gaza Strip, neither of those from the area where the sample was taken. The sample had a high proportion of students from the well-off area (40%), however it includes adolescents from two UNRWA schools; one for boys and one for girls, all having refugee status. Our sample is some what comparable in regard to percentage with resident status, residential area and gender distribution to what has been found in other studies from Gaza [17,41]. The sample was taken from a fairly concentrated area and it consisted of adolescents from a city, a village and a refugee camp (in or close to Gaza city). The main reason was the instability of the political situation during the data collection time, which did not allow for a more representative selection of sample. However, we chose to study the association between sociodemographic factors and meal and food patterns and, not on prevalence of meals and food frequency intakes. We chose strategically to include adolescents from different residential areas and from schools with different providers, as well as including subjects with both refugee and citizen status, in order to secure a wide range of variation in sociodemographic characteristics in the sample. The analyses showed that we had sufficient statistical power to demonstrate sociodemographic differences.

We did not include information about portion sizes in the food frequency questionnaire. We used a list of food items that contained only those most commonly consumed in order to facilitate the data collection. The lack of portion size excludes the possibility of estimating energy and nutrient intakes, however, it has been shown that the frequency intake of a given food item is related to the actual amount of consumed of this item [42]. FFQs are widely used to estimate usual food and it is believed that information captured from food frequency were similar to other dietary data collections tools [43,44]. We consider the use of FFQs without portion sizes to be adequate for the purpose of examining sociodemographic differences.

Under-/over reporting of food intake is common among adolescents [45,46]; therefore, obtaining information on food intakes by self-reporting in children and adolescents is difficult. As previously mentioned, the phenomenon of foods aid provided to the poor families by many charitable organizations may have lead many pupils to under-report their food intakes. Other pupils may want to show a sign of affluence by over-reporting on their food intakes.

The food frequency questionnaire was not validated, but the food list was constructed with a thorough knowledge of the eating pattern in Gaza and with the help of former knowledge about dietary pattern in the West Bank [15]. The lack of data and documentation on adolescents' health in Palestine and in other Middle East countries makes it difficult to compare these results with other findings from the region.

## Conclusion

The meal and food consumption patterns of adolescents in this study are strongly related to socioeconomic status, mothers' level of education and gender. Meal skipping is common, particularly among those of low SES and the intakes of many nutritious foods such as animal food items, fruits and vegetables and foods rich in calcium seem to be low among adolescents of low SES. The results of this study could be used as an important base-line for future monitoring of the nutritional situation of adolescents in Gaza, taking into account the increasing political and economic instability of the territory during the recent years. However, this conclusion could be supported by improved quantitative studies on food intake, including portion sizes. Nutrition programs are needed in schools encouraging sound food habits among adolescents.

## Abbreviations

CHD: Coronary heart disease; CI: Confidence Interval; F/V: Fruits and vegetables; FAO: Food and Agriculture Organization; FFQs: Food frequency questionnaires; MOH: Ministry of Health; OR: Odd Ration; p: Probability; PCBS: Palestinian Central Bureau of Statistics; SES: Household Socio-economic Status; SPSS: Statistical Package for Social Sciences; TV: Television; UNESCO: United Nations Educational, Scientific and Cultural Organization; UNICEF: United Nations Children's Fund; UNRWA: United Nations Relief and Works Agency for Palestine Refugees; VCD: Video CD player; WFP: World Food program; WHO: World Health Organization.

## Competing interests

The authors declare that they have no competing interests.

## Authors' contributions

AHA acquired and analyzed the data and wrote the manuscript; HS joined in with discussion and data analysis; YA contributed to data analysis and to firs draft; ZS contributed to main text and to the final version of the paper; GHO was in charge of the study design and supervised the whole research project. All five authors approved this manuscript.

## Pre-publication history

The pre-publication history for this paper can be accessed here:


